# Role Stress and Prosocial Service Behavior of Hotel Employees: A Moderated Mediation Model of Job Satisfaction and Social Support

**DOI:** 10.3389/fpsyg.2021.698027

**Published:** 2021-09-30

**Authors:** Yixing Jin, Lin Cheng, Ying Li, Yingda Wang

**Affiliations:** School of Tourism, Huangshan University, Huangshan, China

**Keywords:** role stress, job satisfaction, prosocial service behavior, social support, hotel

## Abstract

Hotel employees’ positive behavior is prone to increase customer satisfaction, and thus, exploring the influencing mechanism of role stress on prosocial service behavior is critical to relieving their stress and improving service quality and hotel performance. This study aims to develop and test a moderated mediation model that links hotel employees’ role stress to prosocial service behavior. Based on the conservation of resources theory and job demands-resources model, this study suggests that the effect of role stress on prosocial service behavior is mediated by the level of job satisfaction, whereas the relationship between role stress and job satisfaction is moderated by social support. Data from 256 hotel employees in China largely support the hypotheses that role stress reduces job satisfaction, and that job dissatisfaction is related to low levels of prosocial service behavior. The data also show that job satisfaction partly mediates the relationship between role stress and prosocial service behavior, and social support weakens the relationship between role stress and job satisfaction. The results can help us understand the role of organization-level resources in the workplace and how role stress and job satisfaction affect prosocial service behavior.

## Introduction

In a highly competitive environment, such as the hotel industry, the focus is usually on providing excellent quality service and creating customer satisfaction to obtain and retain loyal customer groups, thereby sustaining competitive advantages (Karatepe and Uludag, 2008; El-Adly, 2019; González-Mansilla et al., 2019; Zhao and Guo, 2019). From the perspective of relationship marketing, improving customers’ cognition and satisfaction with the quality of service depends on establishing and maintaining a long-term harmonious relationship between employees and customers. Specifically, the interaction between hotel employees and customers is an important task and an essential factor to allow customers to perceive, experience, and evaluate the quality of service rendered (Huang and Xie, 2017). Frontline hotel employees play a crucial role in creating positive customer experiences and serve as a reference for customers when evaluating the quality of the service and their satisfaction with it (Elmadağ and Ellinger, 2018). Managers inevitably expect employees to be involved in the daily customer service processes with energetic and positive emotions and a good work attitude to achieve organizational goals. However, in reality, employees suffer heavy stress because of heavy workload, frequently changing working environment, long work hours, poor working conditions with high demands and low resources, and not receiving feedback (O’neill and Davis, 2011; Kim et al., 2012; Karatepe et al., 2018; Khliefat et al., 2021). Role stress is more common, and sometimes inevitable in the hotel industry (Kim et al., 2009). If organizations fail to take effective measures to control employees stress, it may have a negative effect on their job satisfaction, and even trigger burnout (Lambert et al., 2018) and high occurrence of turnover intention (Tongchaiprasit and Ariyabuddhiphongs, 2016), which could affect customers’ perception of service quality.

Most organizational theories and empirical studies focus on the negative effects when discussing role stress, especially studies on the service industry where managers give limited attention to the positive factors (Cameron et al., 2003; Hu et al., 2017). For organizations, while focusing on and addressing problems that develop between employees and the organization is important, the positive factors that promote organizational development should not be ignored because in most cases, people make outstanding achievements when they maximize their strengths instead of focusing on their weaknesses (Buckingham and Clifton, 2001). Therefore, research on role stress should involve not only the negative effects, such as burnout, anxiety, and turnover intention, but also positive outcomes. Prosocial service behavior focuses on the good behavior of employees when providing services to customers or others (co-workers and organizations; Bettencourt and Brown, 1997). Prosocial behavior is an important issue that service marketers and practice managers should be given attention to because it indicates the attitude and behavior of employees that come into contact with customers. Employees’ attitude has a significant effect on how customers evaluate services rendered and customer satisfaction (Alhelalat et al., 2017). Because the essence of service is to help others, enterprises in the hotel industry should exert every effort to improve prosocial service behaviors of employees to promote the formation of harmonious relationships within and outside organizations, which will improve customers’ perception of the quality of service and customer satisfaction (Kang et al., 2020). However, the mechanism involved in the effect of role stress on hotel employees and their prosocial service behavior has not been explained entirely. There is also a relative lack of systematic research on whether role stress directly affects prosocial service behavior, or whether affects it through a change in job attitude or through other moderating factors.

According to the job demands-resources (JD-R) model, excessive job demands (e.g., workload, role conflict, and emotional requirements) can lead to physical and mental exhaustion in employees. However, the provision of significant job resources (e.g., social support, autonomy, and performance feedback) can decrease the demands of the job and the corresponding physical and mental exhaustion and promote personal growth (Demerouti et al., 2001). Social support, as a core element of job resources, is the effective social interaction that an organization provides for employees to motivate them to complete work tasks (Karasek and Theorell, 1990). Social support can prevent and reduce employee stress, help individuals adapt better to changing circumstances and enhance their capacity to overcome setbacks and solve problems (Kwon and Yoon, 2011; Andersén, 2017). In this study, social support is included as a moderator in exploring the effect of the interaction between role stress and social support on job satisfaction and prosocial service behavior.

This study explains the mechanism of how role stress affects attitudes and behaviors of hotel employees from two perspectives, namely job satisfaction and prosocial service behavior. First, attitude toward work can affect the outcome of individuals and organizations. Several studies have confirmed that job satisfaction, as a core element of work attitude, is a useful predictor of job performance, job engagement, organizational citizenship behavior, etc., of employees. Therefore, this study adopts job satisfaction as the mediator variable in explaining the effect of role stress on prosocial service behavior. Second, the availability of social resources can alleviate the negative effects of role stress. In particular, social support behavior in the workplace can help employees deal with stress and negative emotions, thereby improving performance and individual growth. Hence, this study also investigates the moderating effect of social support on the effects of role stress.

## Literature Review

### Role Stress and Prosocial Service Behavior

From the perspective of the organization, role refers to certain behavioral expectations of incumbents of a certain position by role transmitters in the organization (Van Sell et al., 1981). In organizational research, stress that appears in the form of various role-related stressors may engulf resources and create an imbalance between demands and energies (Karatepe and Uludag, 2008). Kahn et al. (1964), a pioneer in the study of role stress, was the first to propose the concept of role stress and defined it as the stress that individuals go through when they are unable to learn or understand the rights and obligations of their work or fulfill their roles well. Sarbin and Allen (1968) claimed that role stress occurs when no specific scope of responsibility is provided or more roles than one can handle, or faces contradictory requirements. When faced with role stress, individuals may experience negative behaviors, such as a decline in performance, job burnout, and may even resort to resigning their post (Wu et al., 2019). Two perspectives can be observed in the study of the dimensions of role stress. One viewpoint claims that role stress can be divided into two dimensions, namely role conflict and role ambiguity (Rizzo et al., 1970; Van Sell et al., 1981). Using in-depth studies on role stress, another viewpoint separates “role overload” from role conflict and defines three types of role stress, namely role ambiguity, role conflict, and role overload (Kahn et al., 1964; Piko, 2006). Some scholars believe that role overload can be divided further into qualitative and quantitative role overloads (Wen et al., 2020). Role conflict originates from inconsistent or incompatible role expectations (Guimaraes and Igbaria, 1992), which occur when work requirements are not consistent with personal standards, values, and work capabilities or when actions contrary to personal values are required. Role ambiguity refers to an individual’s inner feeling when he/she lacks proper understanding of his/her role or fails to obtain definite role expectations (House and Rizzo, 1972). Role overload refers to the stress an individual goes through when he/she fails to meet the role expectations because of the lack of time, energy, or resources (Akgunduz, 2015). Role stress is particularly important in the hospitality industry, where employees are exposed to a variety of service encounters with customers (Brownell, 1990) and often take on multiple and conflicting roles, and serve boundary spanners of the expectations of both the organization and consumers (Kim et al., 2009). When faced with role stress, employees spend more time and energy focusing on task concern and emotion regulation. According to the conservation of resource theory, this process of task concern and emotion regulation caused by external pressure can result in individuals’ internal resource exhaustion, which has serious negative effects on individual and organizational performance.

For a long time, research on employees’ behavior has focused mostly on the behavioral study of internal organizations, such as organizational citizenship behavior (OCB; Ackfeldt and Wong, 2006). However, some types of OCB may be more appropriate for certain sectors than others (Borman and Motowidlo, 1993). Based on the particularity of the service industry, some studies have attempted to clarify the special forms of OCB, such as customer-oriented OCB. Podsakoff and MacKenzie (1997) suggested that other behaviors exhibited by employees to customers, such as prosocial service behavior, should be investigated to improve the understanding of the key driving force of organizational performance. Traditionally, some scholars regard OCB as a prototype of prosocial behavior. However, in terms of its scope, it involves many other types of prosocial behaviors, such as guidance, knowledge sharing, empathy, and compassion (Bolino and Grant, 2016). The term prosocial behavior was first proposed by Wispe (1972), who added the added Latin prefix “pro” meaning “for” to indicate a type of helping behavior. Specifically, prosocial behavior is a behavior or attitude voluntarily shown by individuals for the benefit of others and organizations, without expecting external compensation (Ackfeldt and Wong, 2006; Lacetera and Macis, 2010). Weinstein and Ryan (2010) pointed out that prosocial behavior is not implemented with the expectation of any external factors or rewards but rather involves acting with a positive attitude and helping others and organizations with altruism. Prosocial behavior includes prosocial service behavior and prosocial organizational behavior. Prosocial service behavior is an extension of prosocial organizational behavior, and have the same connotation and essence. However, prosocial service behavior involves mainly feedback from customers, with focus on the helping behaviors of employees to customers and others (co-workers and organizations) in the process of attending to customers (Bettencourt and Brown, 1997). Service-oriented industries, such as hotels, should strengthen the prosocial service behavior of employees because employees’ behavior affects customers’ perception of quality of service and customer satisfaction (Ackfeldt and Wong, 2006; Özduran and Tanova, 2017). From the perspective of service-oriented employees, prosocial service behavior can be divided into role-prescribed customer service, extra-role customer service, and cooperation (Bettencourt and Brown, 1997). Role-prescribed customer service refers to the service behavior expected from service providers based on the job description, evaluation forms, or implicit norms in the workplace (Kang et al., 2020). This behavior can effectively improve customers’ perception of the quality of service (Keaveney, 1995) and improve sales performance (Kelley et al., 1993). Extra-role customer service behavior refers to the act of paying extra attention to customers and providing additional services beyond the formal requirements of the organization (Bettencourt and Brown, 1997). This behavior can impress customers in the process of providing service by showing them inappreciable care, offering exceptional service, providing discount, or through other ways. Cooperation refers to a series of behaviors aimed at helping other employees to provide excellent customer service, thereby creating higher value (Cheng and Chen, 2017). According to the social facilitation theory (Zajonc, 1986), in the service industry, knowledge sharing and tacit cooperation among employees not only improve their work efficiency but also how they cope with external emergencies, thereby improving quality of service, building a smooth service process, and strengthening the positive experience of customers. In the current complex and dynamic business environment, the focus of enterprise marketing has shifted from product to relationship marketing to gain competitive advantages and retain target customer groups. Moreover, the prosocial service behavior of employees plays a key role in promoting the process of building a good relationship with customers.

Existing literature has not yet analyzed the relationship between role stress and prosocial service behavior based on well-defined role stress. However, as a key element of prosocial behavior, OCB has been more widely applied in the research on role stress, and many empirical studies have confirmed the negative effect of role stress on OCB (Kaplan et al., 2009). According to the JD-R model, role conflict leads to exhaustion and burnout among individuals, and this state of internal resource consumption reduces the energy or attention that employees pay to behaviors beyond their formal job description (Schaufeli and Bakker, 2004). Role ambiguity tends to cause uncertainty among employees (Sarbin and Allen, 1968), causing them to consume more resources to seek and obtain information related to their role; thus, they will have less time and fewer opportunities to demonstrate extra-role behaviors (Ladebo, 2006). Employees with clear role definition are more inclined to exhibit discretionary helping behaviors at work (Newman et al., 2015). Only when employees know what their job objectives are and how to achieve them can feel they can achieve service objectives and improve service performance. On the contrary, role ambiguity and role conflict can induce the opposite effect, resulting in the reduction in the occurrence of organizational citizenship and prosocial service behaviors. Moreover, when employees suffer time pressure, they prioritize their job content and pay less attention to the needs of others (Ellis, 2006). People who feel tired because of long hours of work may also be absent from duty frequently, which may reduce the opportunities to implement altruistic behaviors (Jex and Thomas, 2003). Based on the above theoretical analysis, the following hypothesis is proposed:

*H1*: Role stress has a negative effect on employees’ prosocial service behavior.

### Role Stress and Job Satisfaction

Job satisfaction, whether positive or negative, is a subjective attitude formed based on employees’ work experience or assessment of the characteristics of their work (Locke, 1969). Moreover, job satisfaction refers to the level of satisfaction or pleasure that employees gain from the assessment of the work they do or their work experience. Job satisfaction is sometimes measured as the degree to which employees like their work (Spector, 1997). Employees’ evaluation of job characteristics, emotional responses to events that occur in the workplace, and job-related behavior intentions can be used to assess the level of job satisfaction.

The working environment of employees in the hotel industry is irregular and is characterized by diversity and complexity, such as long working hours, role stress, excessive workload, lack of training, unsatisfactory salary, and unpredictable customer behaviors (Karatepe and Kilic, 2007). When individuals are environments that induce stress, they are likely to feel unease and anxiety, causing them to be dissatisfied with work and the development of negative emotions (Tsaur and Tang, 2012). Existing research has shown that role stress has a negative effect on the attitude of employees and enhances individuals’ perception of stress (Stordeur et al., 2001). The most common effect of role stress is the decrease in employees’ job satisfaction. LeRouge et al. (2006) believed that role conflict is the root of job dissatisfaction, tiredness, and absenteeism. Eatough et al. (2011) proved that role stress (role ambiguity and role conflict) has a negative predictive effect on job satisfaction. Kim et al. (2009) conducted a study on hotel employees and found that role conflict and role ambiguity are two important factors that affect job satisfaction and turnover intention of employees. A study based on a group of nurses showed that high role stress experienced by individuals leads to anxiety and dissatisfaction (Kadir et al., 2017). According to the conservation of resources theory (COR), role stress is the process of burning out internal resources that can lead to a change in the attitude of work of individuals, the occurrence of psychological withdrawal, and poor well-being when no effective resource supplementation is available. It can even affect physical and mental health profoundly. Thus, the current study posits that the role stress perceived by hotel frontline employees is likely to decrease their job satisfaction:

*H2*: Role stress has a negative effect on employees’ job satisfaction.

### Job Satisfaction and Prosocial Service Behavior

As a representative of emotional labor, hotel employees’ emotional state directly influences their service behavior and service quality (Wang, 2019). A positive job attitude (job satisfaction) promotes employees’ work engagement, causing them engage in more helping behaviors for consumers. A negative job attitude (job dissatisfaction) reduces positive work experience and the occurrence of out-of-role behavior. Service-oriented organizations always expect employees to show positive customer-oriented behavior. Service-oriented employees treat their customers carefully and kindly and respond effectively to their needs, thereby giving them a better service experience (Lee et al., 2006).

The antecedents and consequences of the prosocial behavior of employees in service marketing in the service industry have been widely discussed in the recent literature. Job satisfaction creates a positive emotional state in the organization that can contribute to social and participatory behaviors (Zare-Khafri and Hassani, 2014). For example, individuals with more job satisfaction in the organization are more likely to display a series of prosocial behaviors similar to OCB (Asgari et al., 2020) and express goodwill and sincerity to customers based on the expectations of the organization because they are expected to repay the organization for treating them well (Wagner and Hollenbeck, 2010). Organ (1988) also indicated that job satisfaction of service providers is an important factor that enhances the citizenship behaviors required to achieve organizational performance, such as helping co-workers and providing additional services to customers. For employees in the service industry in particular, their job satisfaction has a positive effect on their emotional state, and the formation of such positive emotions can promote employees to convey goodwill to customers, thereby creating useful behaviors (George and Brief, 1992). Some of these helping behaviors are implicit or explicit expectations and requirements in the workplace, and some are beyond their job description. Existing research shows that job satisfaction improves employees’ overall service orientation, which is an important contributor to prosocial behavior (MacKenzie et al., 1998). In summary, this study posits that job satisfaction is likely to increase the prosocial service behavior of frontline employees in the hospitality industry. Thus, the following hypothesis is proposed:

*H3*: Job satisfaction of employees has a positive effect on prosocial service behavior.

### Mediating Role of Job Satisfaction

The attitude theory emphasizes that the self-regulation process of an individual can guide his/her behavior (Bagozzi, 1992). An individual’s negative experience can lead to negative responses, which in turn, can prevent employees from achieving personal and career goals (LePine et al., 2005; Malhotra and Ackfeldt, 2016). When employees cannot achieve valuable results at work, they may experience low morale and show less job satisfaction. Long-term exposure to role stress will also make employees feel dissatisfied with their jobs (O’Driscoll and Beehr, 1994), which may also weaken OCB (LePine et al., 2002). Studies have proven the role of job satisfaction as a transmitter of role stress. Singh and Singh (2010) believed that role ambiguity and role conflict affect job satisfaction of employees and that part of the relationships between role ambiguity, role conflict, and OCB might be due to job satisfaction. Bettencourt and Brown (2003) studied the effects of role stressors on customer-oriented boundary-scanning behavior of frontline service employees and found that job satisfaction played a mediating role in the negative effect of role conflict and role ambiguity on customer-oriented boundary-scanning behavior. Goolsby (1992) believed that role stress can be transmitted indirectly to behavior withdrawal response through its effect on psychological withdrawal response. The turnover process of employees is essentially a psychological withdrawal process that can be attributed to reduced job satisfaction and organizational commitment caused by role stress, thereby leading to behavior withdrawal. Ferreira et al. (2017) indicated that job satisfaction not only has a negative effect on the hotel employees’ intention to leave, but also plays a mediating role on turnover intention. Previous studies have confirmed that the turnover behavior of employees is preceded by the work withdrawal behavior of several reduced prosocial organizational behaviors (Chen et al., 1998), indicating that the theoretical model used in explaining employees’ turnover is also conducive for explaining withdrawal behavior from the perspective of prosocial behavior. Thus, we may infer that role stress will negatively influence prosocial service behavior through psychological withdrawal behavior with reduced job satisfaction. Based on the theoretical analysis above, the following hypothesis is proposed:

*H4*: Job satisfaction has a mediating effect on the relationship between role stress and prosocial service behavior.

### Moderating Effect of Social Support

From a psychological perspective, social support refers to the process of experiencing the feelings, which includes the care, response, and facilitation from others (House, 1981). In the work context, social support is defined as the degree of an individual’s perception that their well-being is valued and supported by workplace sources, such as supervisors and co-workers (Eisenberger et al., 2002; Kossek et al., 2011). The positive support of a supervisor can significantly promote employees’ attitude, behavior, and job performance (Amyx and Alford, 2005). A supportive working environment and harmonious working atmosphere among co-workers can help individuals maintain a positive emotional state (Hwa, 2013) and alleviate the negative emotional state of individuals (Duffy and Pagon, 2002). China’s Confucianism advocates “harmony,” which enables Chinese employees to maintain a good relationship with their co-workers and enables co-workers to provide necessary assistance when employees need it (Jin et al., 2014).

Among the job resources included in the JD-R model, social support is considered one of the most important resources for hotel employees. The consumption pathway theory (Demerouti et al., 2001) in the dual pathway of the JD-R model indicates that excessive job demands and the lack of social resources lead to negative organizational outcomes, such as low organizational commitment, job satisfaction, and job performance. As social competition continues to intensify, the work expectations and requirements of organizations are also increasing constantly, and how “consumption” can be transformed to “gains” is a problem faced by every organization. According to the buffer assumption of the JD-R model (Bakker et al., 2005), the abundance of job resources can buffer the consumption of employees caused by job demands. When employees have autonomy, timely feedback, social support, and high-quality superior-subordinate relationship at work, role stress factors, such as job overload and job conflict do not lead to low job satisfaction and a high level of burnout. The buffering effect of job resources can also be explained using the social exchange theory. The formation of a high-level social exchange relationship between the organization and employees leads to the commitment, trust, and loyalty of employees to the organization and induces more behaviors beyond the requirements of external regulations (Hu et al., 2014), that is, employees display more prosocial service behaviors. Supervisors who practice transformational leadership can provide employees with useful feedback and encourage them to make additional efforts to achieve novel solutions, which in turn can enhance employees’ positive work attitude, thereby improving their dedication and prosocial behavior (Gill and Mathur, 2007; Mohamed, 2016). In the COR theory, social support is regarded as an important social resource that can be used to offset the negative effect of the loss of initial resources caused by a stressful work environment (Halbesleben, 2006). Moreover, social support is viewed as a work resource that moderates the effect of job stress on strain (Luk and Shaffer, 2005). Social support in the form of high leader-member exchange (LMX) and mentoring in the workplace can reduce emotional exhaustion because of increased socialization and decreased role stress (Thomas and Lankau, 2009). Previous studies have shown that when employees are in a stressful work environment, support from information resources and emotional support from co-workers can often prevent the formation of negative attitudes (Cohen and Wills, 1985). Hence, this study proposes that social support is likely to moderate the effect of role stress on job satisfaction, as follows:

*H5*: Social support moderates the negative relationship between role stress of hotel employees and their job satisfaction, in that the higher the social support, the weaker the negative relationship between role stress and job satisfaction.

Based on the above theoretical analysis, it can be concluded that social support may also moderate the mediating effect of job satisfaction between role stress and prosocial service behavior. Thus, the indirect effect of role stress on prosocial service behavior through the mediating role of job satisfaction becomes weaker when employees obtain higher social support. By contrast, the indirect effect of role stress on prosocial service behavior through job satisfaction increases when employees receive lower social support. The mediating effect of job satisfaction is moderated by social support, which is a moderated mediating effect. Therefore, based on the mediating role of job satisfaction and the moderating role of social support, a reasonable moderation mediation model is established.

*H6*: The indirect effect of role stress on prosocial service behavior through job satisfaction is moderated by social support, which means that the higher the social support, the weaker the indirect effect of role stress on prosocial service behavior through job satisfaction.

**Figure 1:**
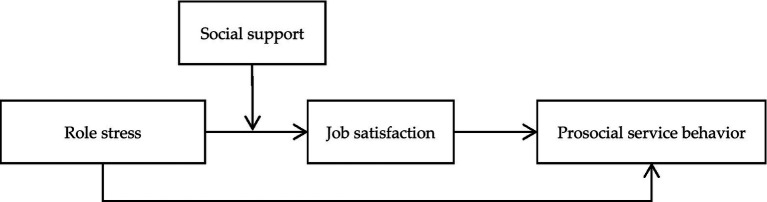
The proposed moderated mediation model.

This study proposes the development of a structural model of role stress, job satisfaction, prosocial service behavior, and social support (Figure 1). The following is our proposed conceptual framework.

## Materials and Methods

### Data Collection

The study conducted convenience sampling of hotel frontline employees. Data collection began on August 24 and ended on September 17, 2020. The respondents were randomly selected from employees of 4- and 5-star hotels in Hangzhou, Beijing, Shanghai, and Huangshan. For the questionnaire survey, the researchers distributed paper questionnaires to frontline employees of the sampled hotels. The questionnaires were placed in sealed envelopes, and the participants were required to hand over the completed questionnaires directly to the researchers to ensure confidentiality and address the concerns of the respondents. A total of 320 paper questionnaires were distributed, and 287 were recovered, indicating a recovery rate of 89.7%. Moreover, after deleting 17 uncompleted surveys and 14 outliers, and finally, a total of 256 questionnaires were used for the empirical analysis. The survey comprised of five items related to the general characteristics of the respondents, including gender, age, education level, tenure, and department. The basic description of the data sample is shown in Table 1.

**Table 1:** Profile of the respondents.

Item	Frequency	Percentage (%)
**Gender (*n* =256)**		
Male	77	30.1
Female	179	69.9
**Age (*n* =256)**		
18–25years	43	16.8
26–30years	63	24.6
31–39years	71	27.7
40–49years	55	21.5
≥50years	24	9.8
**Education Level (*n* =256)**		
Technical school education or below	96	37.5
High school education	69	27.0
College degree	71	27.7
Bachelor’s degree or above	20	7.4
**Tenure (*n* =256)**		
3years and under	120	46.9
4–6years	54	21.1
7–9years	54	21.1
10years and above	28	10.9
**Department (*n* =256)**		
Food and beverage	104	40.6
Front office	28	10.9
Housekeeping	53	20.7
Executive office	13	5.1
Other departments	58	22.7

Table 1 shows the respondents came from different hotel departments, including the food and beverage (40.6%), front office (10.9%), housekeeping (20.7%), executive office (5.1%), and other departments (22.7%). Among the respondents, 69.9 percent of respondents were female and 30.1 percent male. The majority of respondents had finished at most a high school education (64.5%), indicating that the hotel employees are generally not highly educated, and most had between 1 and 9years of job tenure with their hotel (89.1%).

### Measures

#### Role Stress

The role stress scale used in this study is based on the scale developed by Peterson et al. (1995), which was revised to suit the characteristics of Chinese employees by Li and Zhang (2009). This scale is divided into three dimensions, namely role conflict, role ambiguity, and role overload, and assessed with 13 items. A five-point Likert-type response scale was used to measure role stress (from 1=strongly disagree to 5=strongly agree). The Cronbach’s alpha coefficients of the internal reliability of role conflict, role ambiguity, and role overload were 0.800, 0.897, and 0.840, respectively.

#### Job Satisfaction

To test job satisfaction, a separate questionnaire developed by Linz (2003) and used by Torlak and Kuzey (2019) was used in this study. A five-point Likert-type response scale was used to measure the five items of job satisfaction (from 1=strongly disagree to 5=strongly agree). The Cronbach’s alpha coefficient of the internal reliability of job satisfaction was 0.931.

#### Prosocial Service Behavior

This variable was measured with the scale developed by Bettencourt and Brown (1997). The scale is divided into three dimensions, namely extra-role customer service, role-prescribed customer service, and cooperation, and assessed with 15 items. A five-point Likert-type response scale was used to measure prosocial service behavior (from 1=not very true of me to 5=very true of me). The Cronbach’s alpha coefficients of the internal reliability of extra-role customer service, role-prescribed customer service, and cooperation were 0.921, 0.915, and 0.842, respectively.

#### Social Support

This variable was assessed using the dimension of social support included in the job content questionnaire (JCQ; Karasek, 1985), which was translated into the Chinese language. The eight items of the social support scale are distributed into two sub-scales, namely supervisor support (four items) and co-worker support (four items). The questions were answered on a Likert-type scale ranging from 1 to 5 (from 1=strongly disagree to 5=strongly agree). Cronbach’s alpha reliability coefficients of support from supervisor and co-worker were 0.921 and 0.949, respectively.

Table 2 shows that all factor loadings are statistically significant, ranging from 0.660 to 0.899. The CR estimates range from 0.803 to 0.950 (i.e., greater than 0.70). The average variance extracted (AVE) scores of all measurements are greater than 0.50.

**Table 2:** Result of measurement model.

Construct	Items	M	SD	Loading	α	CR	AVE
**Role stress (*n*=256)**					0.905		
Factor 1: role conflict (RC)	RC1	2.336	0.733	0.677	0.8	0.803	0.577
	RC2	2.25	0.703	0.781			
	RC3	2.231	0.739	0.814			
Factor 2: role ambiguity (RA)	RA1	1.984	0.719	0.856	0.897	0.9	0.645
	RA2	2.121	0.81	0.822			
	RA3	2.051	0.658	0.847			
	RA4	2.109	0.743	0.693			
	RA5	1.972	0.641	0.786			
Factor 3: role overload (RO)	RO1	2.059	0.657	0.66	0.84	0.842	0.516
	RO2	2.223	0.698	0.737			
	RO3	2.191	0.69	0.736			
	RO4	2.188	0.728	0.778			
	RO5	2.289	0.769	0.675			
**Job satisfaction (JS; *n*=256)**	JS1	3.481	0.854	0.866	0.931	0.931	0.731
	JS2	3.402	0.885	0.84			
	JS3	3.746	0.827	0.86			
	JS4	3.609	0.809	0.888			
	JS5	3.836	0.79	0.819			
**Prosocial service behavior (*n*=256)**					0.919		
Factor 1: extra-role customer service (ER)	ER1	3.918	0.678	0.775	0.921	0.922	0.703
	ER2	3.922	0.682	0.864			
	ER3	3.93	0.727	0.804			
	ER4	3.938	0.683	0.899			
	ER5	3.973	0.694	0.844			
Factor 2: role-prescribed customer service (RP)	RP1	3.512	0.761	0.829	0.915	0.921	0.699
	RP2	3.516	0.741	0.835			
	RP3	3.593	0.74	0.864			
	RP4	3.27	0.942	0.87			
	RP5	3.148	0.994	0.781			
Factor 3: cooperation (CP)	CP1	3.664	0.67	0.731	0.842	0.843	0.517
	CP2	3.766	0.657	0.692			
	CP3	3.789	0.665	0.722			
	CP4	3.91	0.61	0.722			
	CP5	3.676	0.72	0.728			
**Social support (*n*=256)**					0.958		
Factor 1: supervisor support (SS)	SS1	3.617	0.682	0.836	0.921	0.924	0.752
	SS2	3.664	0.7	0.919			
	SS3	3.688	0.733	0.892			
	SS4	3.59	0.777	0.817			
Factor 2: co-worker support (CS)	CS1	3.723	0.637	0.904	0.949	0.95	0.825
	CS2	3.707	0.642	0.952			
	CS3	3.715	0.655	0.903			
	CS4	3.703	0.69	0.873			

*CR, composite reliability and AVE, average variance extracted*.

### Data Analysis

SPSS 26.0 and Amos 23.0 were applied for data analysis in this study. First, SPSS 26.0 was used to perform descriptive statistics procedures and to test the correlation between variables. Second, Amos 23.0 was used to conduct confirmatory factor analysis (CFA). Third, the four-step procedure suggested by MacKinnon (2008) was adopted to examine the mediation effect of job satisfaction. Finally, Model 7 of PROCESS (Hayes, 2013) was used to examine the moderating effect of social support.

## Results

This study aims to explore whether role stress negatively affects prosocial service behavior, whether job satisfaction mediates the relationship between role stress and prosocial service behavior, and whether the indirect path between role stress and prosocial service behavior is moderated by social support.

### Statistical Description and Correlation Analysis

Table 3 presents the descriptive statistics of the constructs and correlations between the variables. Table 3 shows the statistically significant relationships among role stress, job satisfaction, and prosocial service behavior. Role stress is negatively correlated with job satisfaction and prosocial service behavior (*r*=−0.66, *p*<0.001; *r*=−0.61, *p*<0.001), whereas job satisfaction is positively correlated with prosocial service behavior (*r*=0.60, *p*<0.001). The AVE of each construct exceeds 0.50 and greater than the squared correlation.

**Table 3:** Means, standard deviations, and correlations of observed variables.

Variables	Mean	SD	AVE	1	2	3	4
1. Role stress	2.15	0.49	0.58	**−0.76**			
2. Job satisfaction	3.61	0.74	0.73	−0.66^***^	**−0.85**		
3. Prosocial service behavior	3.7	0.51	0.51	−0.61^***^	0.60^***^	**−0.71**	
4. Social support	3.68	0.61	0.68	−0.52^***^	0.52^***^	0.53^***^	**−0.82**

*** *p<0.001 level*.

*The bold diagonal elements are the square roots of each AVE*.

### Confirmatory Factor Analysis

Before the concrete hypothesis verification, confirmatory factor analysis was used to examine the validity of the variables. The structural model has the following factors: three dimensions of role stress, three dimensions of prosocial service behavior, two dimensions of social support, and job satisfaction. The conceptual model of the study has good model fit [*x*^2^(743)=1301.57, *p*<0.001; *x*^2^/*df*=1.75; GFI=0.81; TLI=0.93; CFI=0.93; RMSEA=0.05].

### Mediating Effect of Job Satisfaction

H4 assumes that job satisfaction mediates the relationship between role stress and prosocial behavior. To examine this hypothesis, this study adopts a four-step procedure to establish the mediation effect (MacKinnon, 2008), including (a) a significant relationship between role stress and prosocial service behavior, (b) a significant relationship between role stress and job satisfaction, (c) a significant relationship between job satisfaction and prosocial service behavior while controlling for role stress, and (d) a significant coefficient for the indirect path between role stress and prosocial service behavior through job satisfaction. The bias-corrected percentile bootstrap method was used to examine whether the last condition is satisfied.

According to the results of the multiple regression analysis in Table 4, in the first step (Model 1), role stress is significantly related to prosocial service behavior (*b*=−0.61, *p*<0.001), and thus, H1 is supported. In the second step (Model 2), role stress is significantly related to job satisfaction (*b*=−0.66, *p*<0.001), which supports H2. In the third step (Model 3), when role stress was controlled, job satisfaction remained significantly related to prosocial service behavior (*b*=0.33, *p*<0.001), and thus H3 is supported. Finally, Table 5 shows that based on the bias-corrected percentile bootstrap method, the indirect effect of role stress on prosocial service behavior through job satisfaction is significant (ab=−0.22, SE=0.05, 95% CI=[−0.33, −0.12]). The indirect effect accounts for 36.07% of the total effect. Overall, the four criteria for establishing the mediation effect are satisfied, thereby supporting H4.

**Table 4:** Mediation effect of job satisfaction.

Predictors	Model 1 (PB)		Model 2 (JS)		Model 3 (PB)	
	*b*	*t*	*b*	*t*	*b*	*t*
Gender	−0.04	−0.08	0.03	0.51	−0.01	−0.26
Age	0.09	1.72	0.08	1.65	0.06	1.25
Education Level	0.06	1.09	0.04	0.72	0.05	0.91
Tenure	0.04	0.68	0.01	0.18	0.03	0.66
Department	0.01	0.17	0.01	0.24	0.01	0.1
RS	−0.61	−11.99^***^	−0.66	−13.68^***^	−0.39	−6.08^***^
JS					0.33	5.27^***^
*R* ^2^	0.39		0.44		0.45	
F	26.25^***^		33.08^***^		28.88^***^	

*** *p<0.001 level*.

*RS, role stress; JS, job satisfaction; and PB, prosocial service behavior*.

**Table 5:** Total effect, direct effect, and indirect effect in study model.

	Effect	Boot SE	Boot LLCI	Boot ULCI	Proportion of effect
Total effect	−0.61	0.05	−0.73	−0.48	
Direct effect	−0.39	0.07	−0.52	−0.24	63.93%
Indirect effect	−0.22	0.05	−0.33	−0.12	36.07%

### Moderating Effect of Social Support

In this study, H5 predicts that social support moderates the relationship between role stress and job satisfaction, and H6 predicts that the indirect relationship between role stress and prosocial service behavior is also moderated by social support (Figure 1). We adopt Model 7 of PROCESS developed by Hayes (2013) to examine the moderating effect of social support.

**Figure 2:**
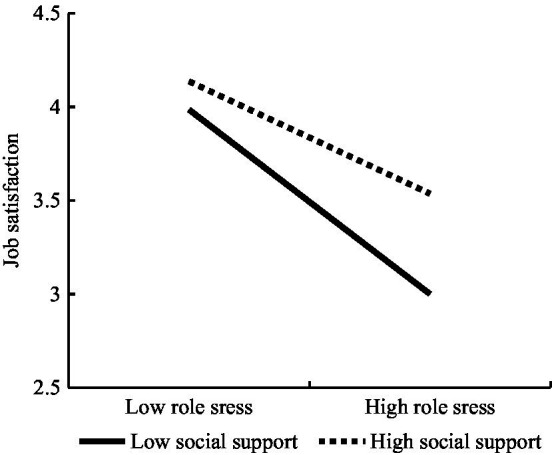
The interaction between role stress and social support on job satisfaction.

Table 6 demonstrates the significant effect of role stress on job satisfaction (*b*=−0.54, *p*<0.001), and this effect is moderated by social support (*b*=0.13, *p*<0.001). For descriptive purposes, this study plotted the predicted job satisfaction against role stress for low and high levels of social support separately. Figure 2 shows that role stress is more negatively related to job satisfaction when social support is low (*b*=−0.67, 95%CI=[−0.80, −0.54]) than when it is high (*b*=−0.40, 95%CI=[−0.53, −0.28]), and thus, H5 is supported.

**Table 6:** Moderated regression analysis.

Predictors	Job satisfaction		
	SE	*b*	*t*
Gender	0.10	0.03	0.33
Age	0.04	0.06	1.50
Education Level	0.05	−0.01	−0.05
Tenure	0.04	0.02	0.54
Department	0.03	0.03	1.04
RS	0.05	−0.54	−10.16^***^
SS	0.05	0.24	4.54^***^
RS*SS	0.04	0.13	3.42^***^
*R* ^2^	0.51		
∆R^2^	0.02^***^		
F	32.21^***^		

*** *p<0.001 level*.

*RS, role stress and SS, social support*.

The bias-corrected percentile bootstrap method with SPSS further indicates that the indirect effect of role stress on prosocial service behavior through job satisfaction is moderated by social support. Table 7 shows that for social support, the indirect effect of job satisfaction is stronger and significant in a low social support condition (*b*=−0.22, Boot 95% CI=[−0.33, −0.11]), but is weaker and significant in a high social support condition (*b*=−0.13, Boot 95%CI=[−0.22, −0.07]). Finally, the index of moderated mediation (Index=0.04, Boot 95%CI=[0.01, 0.08]) indicates that the moderated mediation effect is significant, thereby supporting H6.

**Table 7:** Bootstrap test of the moderated mediation effect.

Mediator	Social support	Effect	Boot SE	Boot LLCI	Boot ULCI	Index
Job satisfaction	M−1SD	−0.22	0.06	−0.33	−0.11	0.04 [0.01, 0.08]
	M	−0.18	0.04	−0.27	−0.09	
	M+1SD	−0.13	0.04	−0.22	−0.07	

## Discussion

This study explores the mechanism of the effect and boundary conditions of role stress on the prosocial service behavior of hotel employees in china from the perspective of the COR theory. A conceptual framework was proposed to uncover the prosocial service behavior of hotel employees by investigating the effects of role stress. This study also investigates the mediating effect of job satisfaction on role stress and prosocial service behavior, and the moderating effect of social support on role stress and job satisfaction. The findings support the proposed hypotheses. The conclusions are as follows: (1) role stress has a direct negative effect on job satisfaction and prosocial service behavior, (2) job satisfaction not only has a direct effect on prosocial service behavior, but it also plays a mediating role between role stress and prosocial service behavior, and (3) social support plays a moderating role between role stress and job satisfaction, and social support moderates the mediating effect of job satisfaction.

### Theoretical Implications

This study provides important insights into the subject of the prosocial service behavior of frontline hotel employees in China. The research findings offer significant theoretical contributions in the construction of the theoretical framework for prosocial service behavior. Previous studies have shown that from the perspective of negative outcomes, role stress leads to frustration, burnout, and work–family conflict, which can also result in more adverse consequences, such as absenteeism and high employee turnover (Chen and Spector, 1992; Stoeva et al., 2002; Jung et al., 2012). From the perspective of positive outcomes, role stress can decrease employees’ job satisfaction, OCB, and altruistic behavior, and even affect employees’ life satisfaction and happiness (Duygulu et al., 2013). This study focuses on the influence mechanism of role stress on employees’ positive behavior. Prosocial service behavior is an important outcome variable in positive organizational scholarship, which has been redefined as a compatible construct for service sectors (Bettencourt and Brown, 1997). In the hospitality industry, exploring the influence of role stress on customer-oriented prosocial behavior has considerable significance. The following theoretical implications are proposed.

First, this study reveals that role stress of individuals is an important determinant of less prosocial service behavior. Role stress of frontline hotel employees in the working environment can lead to depression and emotional dissonance (Schmidt et al., 2014). Employees with greater degree of role stress usually lack job motivation, making them less willing or able to exhibit high job performance (Akgunduz, 2015). Prosocial service behavior implies that frontline employees should not only focus on their role but also provide extra-role services for customers and also have the time and energy to actively help co-workers. Role conflict and role ambiguity causes employees to be confused and renders them unable to focus effectively on the requirements of the organization, co-workers, and customers. Role overload can result in physical and mental exhaustion of employees, which leaves them with no time and energy to carry out extra-role and mutual assistance behaviors. According to the primary principle for resource loss in the COR theory, individuals might be inclined to adopt the resource conservation principle to avoid resources losses caused by role stress, which leads to the psychological withdrawal of employees toward work and organizations (e.g., dissatisfaction with work and reduced organizational commitment), and even behavioral withdrawal of employees (e.g., reduced prosocial service behavior and turnover; Anton, 2009). The results of this study further demonstrate the negative effect of role stress on employees’ positive behavior.

Second, this study explores the relationship between role stress and prosocial service behavior of hotel employees, and examines it by setting job satisfaction as a mediating variable. Employees spend more time and energy on position-related information to fulfill task requirements when faced with role stress. This consumption process will inevitably lead to a reduction in the attention paid to customers, thereby reducing prosocial service behavior. Employees who experience long-term role stress will be more likely to feel dissatisfied with their jobs (Kim et al., 2009) and reduce their prosocial service behavior. According to the COR theory, challenging and hindering stressors are job demands. To meet the job demands, employees need to consume a certain amount of resources and energy, which leads to negative emotional reactions before resources are compensated, which could further lead to job burnout after long-term accumulation. Job dissatisfaction due to role stress will trigger burnout, which will further affect work behavior. Previous studies have shown that the relationship between role stress and task performance is affected by job satisfaction (Fried et al., 2008). This study demonstrates that job satisfaction is not only related closely to prosocial service behavior but also transmits the negative effect of role stress.

Third, social support moderates not only the effect of role stress on job satisfaction but also the mediating effect of job satisfaction. Extensive theoretical support for the effect of supportive factors in the work environment in relieving employees’ role stress can be found. A supportive work environment stems from the high involvement and assistance from co-workers in the workplace and the support and facilitation from supervisors (Babin and Boles, 1996). In the context of Chinese culture, employees with a strong tendency toward collectivism will emphasize the maintenance of group harmony (Walumbwa and Lawler, 2003). The care, approval, and respect from supervisors and co-workers can fill the socio-emotional needs of Chinese employees, thereby strengthening their affective commitment and positive behavior (He et al., 2011). In a high-quality LMX relationship, the emotional and work supportive behavior of supervisors can help employees to clearly understand the scope of their work in the organization, clarify their job responsibilities, and set up correct work objectives, thereby alleviating the negative effect of role stress on employees (Thomas and Lankau, 2009). Moreover, some studies have shown that social support from co-workers effectively reduces the effects of workload on job stress by helping employees complete job tasks on time (Van der Doef and Maes, 1999). The results revealed that sufficient supportive social resources in an organization’s environment enable employees to deal with stressors more effectively, fulfill work objectives, and improve job satisfaction, thereby affording employees more opportunities to perform prosocial service behaviors.

### Managerial Implications

Service is an experiential intangible product. Customers not only pay attention to the perfection and optimization of hardware facilities but also care more about the emotional interaction and experience when purchasing a service. Frontline employees in the service industry play an important role in providing excellent service to customers and improving service performance (Babakus et al., 2003). Therefore, to gain sustainable service advantages, the hotel industry should strive to remove the negative factors that affect the work attitudes and behaviors of employees, enhance employees’ job satisfaction, and reinforce employees’ prosocial service behaviors. Although previous academic discussions have centered on the mechanisms of the challenges and hindrance of stress, undoubtedly, individuals cope with role stress by burning out their internal resources. According to the affective event theory, stressors in the workplace, whether challenging or hindrance, can be regarded as the affective events that lead to employees’ affective reactions (Rodell and Judge, 2009). Amah (2014) indicated that challenging and hindrance stressors can weaken employees’ job satisfaction. Managers are faced with the problem of how they can help employees maintain good mental health in a complex service environment. Based on the results of this study, the following managerial implications have been proposed:

First, hotel management should focus on how it can effectively reduce the occurrence of role stress of employees, relieve their existing stress, and motivate them to actively provide services. Currently, frontline employees in the hotel industry are under stress from various sources, such as the demands of the organization, the increasing expectations of customers, and even work–family conflicts. The role stress of frontline employees increases during tourism seasons in particular. Hence, hotel management should determine and understand how employees can maintain prosocial behavior in high-intensity work. Organizations should give priority to stressors, such as role ambiguity. An effective strategy for enhancing employees’ prosocial service behavior is to set clear role expectations. Clear role orientation can minimize role stress to a high degree and help employees provide customers with higher levels of service (Mukherjee and Malhotra, 2006). To achieve this goal, hotel managers should improve role clarity of employees through training, information sharing, constituting mutual assistance teams, and creating a learning atmosphere. Hotel management should also reasonably arrange employees’ work tasks, avoid giving employees contradictory work requirements and assist employees in dealing with the different requirements of customers. Moreover, managers should also focus on the emotional state of employees, create a good working atmosphere, and provide guidance and training to enable employees to obtain the means and skills required to alleviate role stress.

Second, the negative effect of role stress on prosocial service behavior is verified in this study, and more importantly, this negative effect is mediated by job satisfaction. This mediating role gives managers the insight that role stress of employees is not a direct factor contributing to reduced prosocial behavior. Role stress may cause employees to feel unequilibrated in many aspects, but the degree of job dissatisfaction caused by role stress is the main cause of reduced prosocial behavior of employees. The results of this study indicate that the negative effect of role stress on job satisfaction is significant. Hence, the findings of this study also correspond to those of previous studies (Schaubroeck et al., 1989; Örtqvist and Wincent, 2006). Therefore, while alleviating role stress of employees, managers should also focus on the changes in employees’ job satisfaction, and implement effective intervention measures to improve the job satisfaction of employees, thereby realizing the changes in employees’ attitude and behavior in the process of serving customers. Admittedly, reducing the degree of role stress can reduce employees’ job dissatisfaction through effective management measures, but because of the particularity of the service industry, employees’ role stress in the workplace is inevitable (Kim et al., 2009). Therefore, to manage role stress, organizations should reduce the negative effects of role stress on job satisfaction through job rotation (De Ruyter et al., 2001), the provision of fitness facilities, improvement of welfare treatment, and stress consultation to help employees to implement prosocial service behavior with full enthusiasm.

Finally, the results show that the moderating effect of social support is significant. In reality, as employees have to be dependent on the supervisors who are high in power and status (Gordon et al., 2019), the supervisors play a vital role in inducing employees to exhibit helping behavior (Hai and Park, 2021). If employees feel that supervisors care about their needs and are willing to provide them with emotional support, they will have a positive evaluation of their working environment and display higher job satisfaction (Kopelman et al., 1990). Supervisors can likely make employees who experienced stress at work to become more creative by providing positive and useful feedback to employees (Hon et al., 2013). In addition, the relationships of mutual assistance with co-workers also contribute to a positive job experience of employees. A formal support group can create organizational climates that cushion employee stressors related to socio-emotional work (Pecino et al., 2019). Therefore, organizations should alleviate organizational constraints by establishing supportive work environments in which employees are given the necessary tools, technology, and information to function effectively (Gilboa et al., 2008). In management practices, managers should not only establish a work allocation system that matches the abilities of individuals but also prepares employees to actively cope with the negative effects of role stress through effective social support. The means of support should not only be limited to the working conditions and information support but also include emotional support. In short, an open and smooth communication channel should be formed within the organization, and continuous attention should be paid to employees in real time. The organization can help employees to actively face role stress, reduce emotional exhaustion, and promote prosocial service behavior by building harmonious, friendly, and mutually supportive interpersonal relationships.

### Limitations and Directions for Future Research

In this study, the questionnaire survey was conducted only on hotels in Eastern China because of limited time, resources, geography, and other conditions. Thus, the sample used may lack representativeness. Considering the differences in regions on economic, cultural, and conceptual levels, the sample size should be expanded to focus on hotels in different regions in future research.

Second, a cross-sectional questionnaire survey method was used in this study, and the variables were measured based on the same time node, leading to insufficient causal inference between variables. Thus, in future research, a more detailed demonstration could be obtained through a longitudinal research design.

Third, this study utilized the averaging item method to combine the items in the same factor, and eventually used a total score to explore the relationship between role stress and other variables. Therefore, in order to avoid the loss of information that may be caused by the averaging item technique, it is necessary for future research to dig deeply into the relationship between each sub-dimension, and explore deeply the mediating role of job satisfaction and the moderating role of social support in the model.

Finally, in this study, only social support was set as the moderating variable, but many factors can alleviate the negative effects of role stress. In future research, diverse moderating variables can be extracted from empirical research from multiple perspectives, such as individual characteristics, organizational environment, and family factors.

## Conclusion

The results show that the model of this study is applicable to frontline employees of hotels in Eastern China. This study investigated a moderated mediating process from role stress to prosocial service behavior and found that (1) high role stress in the workplace will reduce the prosocial service behavior of employees by reducing their job satisfaction and (2) social support will buffer the negative effect of role stress on job satisfaction and moderate the mediating role of job satisfaction in the negative relationship between role stress and prosocial service behavior. In conclusion, this study is expected to fill a void in the literature concerning positive behavior studies in the hospitality field.

## Data Availability Statement

The raw data supporting the conclusions of this article will be made available by the authors, without undue reservation.

## Author Contributions

YJ and YL conceptualized the research. YJ and LC wrote the manuscript. LC and YW collected and analyzed the data, which were analyzed by YJ and YW. All authors read and approved the manuscript, and agreed to be accountable for all aspects of the work.

## Funding

This research was supported by the Humanities and Social Science Project of the Higher Education Institutions of Anhui Province, nos. SK2016A0883 and SK2019A0429.

## Conflict of Interest

The authors declare that the research was conducted in the absence of any commercial or financial relationships that could be construed as a potential conflict of interest.

## Publisher’s Note

All claims expressed in this article are solely those of the authors and do not necessarily represent those of their affiliated organizations, or those of the publisher, the editors and the reviewers. Any product that may be evaluated in this article, or claim that may be made by its manufacturer, is not guaranteed or endorsed by the publisher.
